# The Intestinal Carriage of Plasmid-Mediated Colistin-Resistant Enterobacteriaceae in Tertiary Care Settings

**DOI:** 10.3390/antibiotics10030258

**Published:** 2021-03-04

**Authors:** Jan Tkadlec, Alzbeta Kalova, Marie Brajerova, Tereza Gelbicova, Renata Karpiskova, Eva Smelikova, Otakar Nyc, Pavel Drevinek, Marcela Krutova

**Affiliations:** 1 Department of Medical Microbiology, 2nd Faculty of Medicine, Charles University, 150 06 Prague, Czech Republic; Marie.Brajerova@fnmotol.cz (M.B.); EvaSmelikova@seznam.cz (E.S.); Otakar.Nyc@fnmotol.cz (O.N.); pavel.drevinek@lfmotol.cuni.cz (P.D.); marcela.krutova@lfmotol.cuni.cz (M.K.); 2 Department of Medical Microbiology, Motol University Hospital, 150 06 Prague, Czech Republic; 3 Department of Microbiology and Antimicrobial Resistance, Veterinary Research Institute, 621 00 Brno, Czech Republic; gelbicova@vri.cz (T.G.); karpiskova@vri.cz (R.K.); kalova@vri.cz (A.K.); 4 Department of Experimental Biology, Faculty of Science, Masaryk University, 611 37 Brno, Czech Republic

**Keywords:** *E. coli*, colistin, mcr, Czech Republic, IncX4, silent carriage

## Abstract

**Background:** In order to estimate the prevalence of plasmid borne colistin resistance and to characterize in detail the *mcr*-positive isolates, we carried out a sentinel testing survey on the intestinal carriage of plasmid-mediated colistin-resistant Enterobacteriaceae in hospitalized patients. **Methods:** Between June 2018 and September 2019, 1922 faecal samples from hospitalised patients were analysed by selective culture in presence of colistin (3.5 mg/L), and in parallel by direct detection of the *mcr-1* to *mcr-8* genes by qPCR. The *mcr*-positive isolates were characterised by whole-genome sequencing. **Results:** The prevalence of the *mcr-1* gene was 0.21% (*n* = 4/1922); the *mcr-2* to *8* genes were not detected. The *mcr-1* gene was found to be localised in the IncX4 (*n* = 3) and IncHI2 (*n* = 1) plasmid type. One *Escherichia coli* isolate was susceptible to colistin due to the inactivation of the *mcr-1* gene through the insertion of the IS2 element; however, the colistin resistance was inducible by culture in low concentrations of colistin. One human *mcr-1* positive *E. coli* isolate was related genetically to the *mcr-1 E. coli* isolate derived from turkey meat of Czech origin. **Conclusions:** *mcr*-mediated colistin resistance currently poses little threat to patients hospitalised in Czech healthcare settings. The presence of the *mcr-1* gene in the human population has a possible link to domestically produced, retail meat.

## 1. Introduction

Colistin is a polypeptide antibiotic belonging to the polymyxins. The outer membrane is a site of polymyxin interaction, which makes them effective against most Gram-negative bacteria [1]. However, due to its nephrotoxicity, colistin has had a limited use in human medicine; yet now, with the growing threat of the antimicrobial resistance, colistin has emerged as one of the last-resort antimicrobials for the treatment of infections caused by multidrug-resistant Gram-negative pathogens [2]. The resistance of Enterobacteriaceae to colistin began to be studied intensely following the emergence of plasmid-mediated colistin resistance encoded by the *mcr-1* gene in China in 2015 [3]. To date, another nine *mcr*-genes have been identified [4], with *mcr*-10 being the latest [5]. However, the *mcr-1* gene is the most frequently detected; yet the number of studies on the prevalence of the *mcr-2* to *mcr-10* genes is limited [6].

The occurrence of *mcr*-mediated colistin resistance is widespread, has been detected on all continents of the world, except for Antarctica, and has a variety of sources (human, animals, food of animal origin and the environment) [6]. Livestock, poultry and related food products have been identified as a reservoir for the spread of *mcr* genes carrying Enterobacteriaceae into humans [3]. The high prevalence of *mcr*-mediated colistin resistance in livestock has been linked to the extensive use of colistin in the veterinary sector and, when compared to human medicine, colistin has been used extensively for decades in the prevention and treatment of enterobacterial infections as well as a growth promoter [1,7]. While in Europe the veterinary use of polymyxins is monitored and regulated [8], in Asia, however, no such regulations exist, so colistin is produced and used predominantly [3]. The relationship between the agricultural use of colistin and the spread of plasmid-mediated colistin resistance was demonstrated by a Chinese study that detected a significant decrease (37% to 1%) of *mcr* gene prevalence among hospitalized patients after a ban on the agricultural use of colistin [9]. In addition, the intestinal carriage of *mcr-1* was reported to have a high prevalence among people from rural areas in Bolivia (38.3%) [10] and Vietnam (20.6% and 65.31%, respectively) [11,12].

In Europe, the available data show that the prevalence of *mcr-1* intestinal carriage in hospitalized patients is <1% [13,14,15,16,17]; however, in the community, the prevalence of *mcr*-gene carrying Enterobacteriaceae was found to be higher (4.9% and 11.4%) in cohorts of German and Dutch travellers [18,19].

Recently, Enterobacteriaceae with plasmids carrying the *mcr-1* gene were detected in as much as 70% of tested retail meat samples in the Czech Republic [20], pointing to a possible source of colistin resistance, but little is known about the prevalence of the *mcr*-genes in humans. We aimed to estimate the prevalence of intestinal carriage of colistin-resistant Enterobacteriaceae with a detailed characterisation of the isolates carrying the *mcr* genes in patients of a large tertiary care hospital in the Czech Republic.

## 2. Results

### 2.1. The Prevalence of the mcr-1 Gene Was Low, and the mcr-2 to -8 Genes Were Not Detected

Between June 2018 and September 2019, 1922 samples (1657 rectal swabs and 265 stool samples) were investigated. Average age of the patient was 30 years (SD 29.06; range 1 day to 96 years), and 53.43% of the patients were males. The *mcr-1* gene was detected in four enriched cultures; the prevalence of *mcr-1* gene carriage was 0.21%. The *mcr-2* to *-8* genes were not detected.

After culture of the enriched cultures on chromogenic selective agar supplemented with colistin, 294 isolates of the Enterobacteriaceae were acquired, and the colistin resistance was confirmed in 131 isolates using the broth microdilution method; the prevalence of the gut carriage of colistin-resistant Enterobacteriaceae was 6.82% (Table 1).

In colistin-resistant Enterobacteriaceae, three *mcr-1* positive *E. coli* were identified. Two of the *mcr-1* positive isolates corresponded to the *mcr-1* PCR-positive enrichment cultures. An additional *mcr-1* positive *E. coli* strain was cultured from a PCR-negative enrichment culture.

The remaining two *mcr-1* positive enrichments, with a subsequent negative culture on chromogenic selective media supplemented with colistin, were inoculated onto chromogenic media without colistin; one *mcr-1* positive *E. coli* isolate (P1301A) was detected and found to be susceptible to colistin (MIC = 0.25 mg/L). The chromogenic culture-based prevalence of *mcr-1* positive colistin resistant *E. coli*, after an enrichment and culture on selective media supplemented with colistin, was 0.16%. When the *mcr-1* positive/colistin- susceptible isolate P1301A was included, the chromogenic culture-based prevalence of *mcr-1* positive *E. coli* increased to 0.21%.

The production of MCR-1 phosphoethanolamine transferase was confirmed by lateral flow immunoassay in all of the colistin-resistant *mcr-1* positive *E. coli* isolates (*n* = 3) except for one colistin-susceptible *mcr-1* positive isolate (P1301A).

The MICs of other antimicrobials tested against the *mcr-1* positive *E. coli* isolates are shown in Table 2.

Resistance mechanisms in *mcr-1* to *mcr-8* negative isolates were not studied.

### 2.2. Whole-Genome Analysis Data Revealed a High Diversity of mcr-1 Positive E. coli Isolates and the Predominance of the IncX4 Plasmid Type as a mcr-1 Gene Vector

The *mcr-1* positive *E. coli* isolates belonged to four distinct sequence types, and cgMLST analysis revealed a high diversity of *mcr-1* isolates in our study (Table 3). In silico bioinformatic analysis showed the corresponding acquired resistance genes or chromosomal mutations associated with the resistance phenotype, except for the colistin susceptible-*mcr-1* positive isolate (Table 4).

The *mcr-1* positive isolates carried multiple plasmids (Table 4). The *mcr-1* gene was localised on the plasmid type IncHI2 (250 kb) in isolate P1519A, together with additional antimicrobial resistance genes (*aadA1*; *aadA2b*; *aph*(3′)-Ia; *cmlA1*; *tet*(A); and *sul3*). In another three isolates, the *mcr-1* gene was localised in the IncX4 plasmids of 33–34 kb size, and no other resistance gene was found in that plasmid (Table 4).

**Figure 1:**
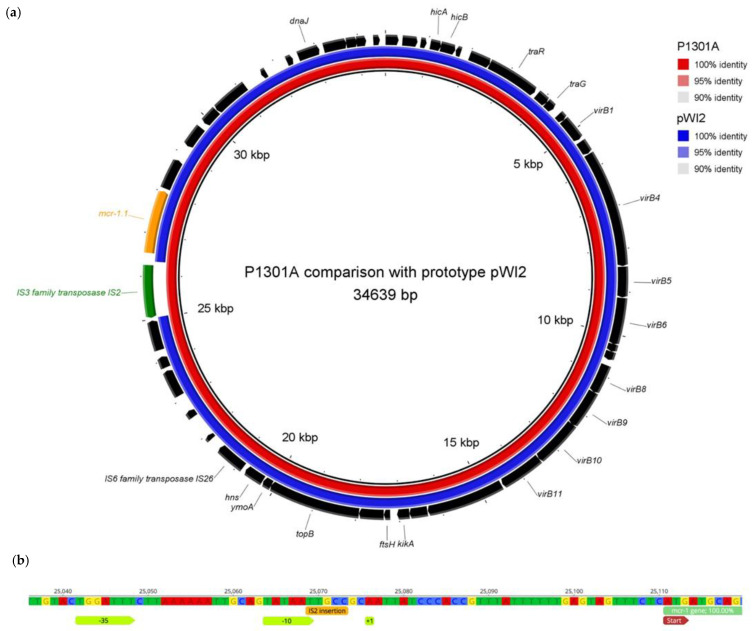
Genetic cause of colistin susceptibility in *mcr-1* positive *Escherichia coli* isolate P1301A. (**a**) A comparison of the prototypic *mcr*-bearing IncX4 plasmid pWI2-*mcr* and a plasmid from the *mcr-1* positive/colistin-susceptible *E. coli* isolate (P1301A). The plasmids were compared using a BRIG (Blast Ring Image Generator) v0.95, and annotation of the genes was done by Prokka v1.14.5 and RAST software (https://rast.theseed.org/FIG/rast.cgi, accessed on 10 November 2020). Hypothetical proteins not shown (Supplementary Table S1). (**b**) Details of the IS2 element insertion site in the promoter region of the *mcr-1* gene of the prototypic pWI2-*mcr* plasmid. The insertion of the IS2 element into the IncX4 plasmid of the P1301A isolate lead to a separation of the -10 and +1 promoter regions and a silencing of *mcr-1* expression. The sequence of the *mcr*-promoter was derived from the study of Poirel et al. [24].

To assess the diversity of the IncX4 plasmids detected in this study, the sequences of the plasmids were compared to a prototypic IncX4 plasmid pWI2-*mcr* (33,304 bp) available in the NCBI database (accession no LT838201) [23]. Using a MUSCLE alignment, the plasmid from P642A isolate (33,308 bp) showed a 99.99% identity to pWI2-*mcr*, for plasmids from the P732A (34,080 bp) and the P1301A (34,639 bp) isolates the identity was 100% with 97.7% and 96.1% coverage, respectively. The differences in the size of these plasmids compared to the prototypic pWI2-*mcr* were caused by the presence of insertion sequences in close proximity to the *mcr-1* gene. In the isolate P732A, the IS*1R* (776 bp) element belonging to the IS1 family was inserted immediately before -35 position of the *mcr* promoter region. In the colistin-susceptible isolate P1301A, the promoter region of the *mcr-1* gene was disrupted by the IS2 element (1336 bp) that separated the -35 and -10 boxes from the translation start (Figure 1).

Fasta files of hybrid assemblies for the *E. coli* isolates P642A; P732A; P1301A and P1519A are available at Mendeley data: doi: 10.17632/9pwrnrnx2n.1, accessed on 10 November 2020).

### 2.3. Colistin Resistance in the mcr-1 Positive/Colistin-Susceptible Isolate Was Inducible

To test the inducibility of colistin resistance in the isolate P1301A, we cultured the isolate in LB broth supplemented with colistin in concentrations ranging from 0.064 to 1 mg/L overnight at 37 °C. Bacterial growth was visible in all colistin concentrations tested, and using a lateral flow immunoassay, the presence of MCR-1 phosphoethanolamine transferase was detected in cultures with concentrations of colistin at 0.25 mg/L and higher.

Subsequent antimicrobial susceptibility testing of colistin showed an increase in MICs that exceeded the breakpoint of 2 mg/L up to 8 mg/L in isolates grown in concentrations of 0.25 mg/L and higher. In isolates grown in concentrations of 0.064; 0.125 mg/L and in controls cultured without colistin the MICs to colistin remained unchanged (0.25 mg/L).

To investigate the mechanism of the inducibility of colistin resistance in the isolate P1301A, the primers mcr1_137_F: CAGTATGGGATTGCGCAATGA and mcr1_891_R: AAGAAAACGGCAACACTCGC (Supplementary Table S3) were designed to amplify a region upstream of the *mcr-1* containing promoter that controls MCR-1 production. Sanger sequencing of the PCR product was performed in subcultures of isolate P1301A where the colistin resistance was induced; the concentration of colistin in cultures was 0.25 mg/L and higher. Compared to the WGS data acquired from the same isolate, and before the induction of colistin resistance, the precise excision of IS2 without a target site duplication was found. The perfect restoration of the wild-type promoter region of the *mcr-1* gene was the cause of the colistin resistance.

### 2.4. The Human mcr-1 E. coli Isolate Was Genetically Related to the mcr-1 E. coli Isolate from Turkey Meat Produced Domestically

**Figure 2:**
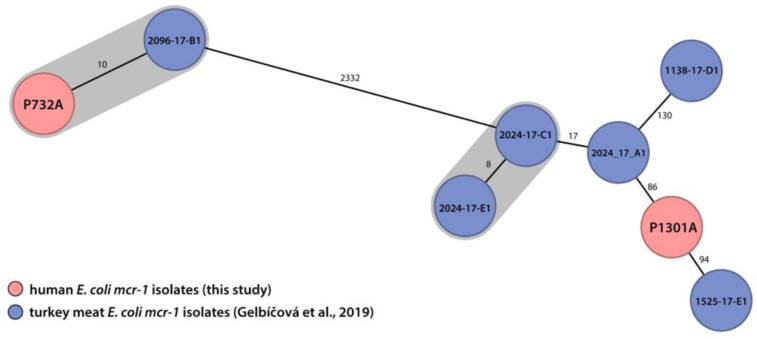
A comparison of the minimum spanning tree using a core genome MLST (cgMLST) analysis comprising 2513 loci for *E. coli*.

Two human *mcr-1 E. coli* isolates (P732A, P1301A) were compared with six *mcr-1 E. coli* isolates derived from turkey meat [25] that have the same ST (744 or 69) and carry the *mcr-1* gene in the same plasmid type (IncX4). Surprisingly, one human isolate (P732A) was related genetically to the *mcr-1 E. coli* isolate derived from domestically produced turkey meat (2096-17-B1) as demonstrated by a 10 allele difference in the 2513 loci of the cgMLST. Their genetic relatedness is also supported by the similarity of their resistance profiles at a phenotypic and genotypic level (Figure 2). The second human isolate (P1301A) demonstrated an 86 allele difference to the closest turkey-meat isolate excluding their close genetic relatedness. A close genetic relatedness was not found using the BacWGSTdb 2.0 database. The search results are shown in Supplementary Table S2.

## 3. Discussion

To the best of our knowledge, this is the first study to detect the human intestinal carriage of Enterobacteriaceae possessing the *mcr-1* to *-8* genes.

Compared to other studies that screened hospitalised patients, the prevalence of the *mcr-1* gene in our study (0.21%) was found to be lower than that from study in Chinese tertiary hospitals in 2016, where the prevalence was 37.1% in 832 asymptomatic inpatients, and, in 2018, following a ban on the agricultural use of colistin, it was 1.38% in 654 inpatients [9]. An additional Chinese study reported that the prevalence of *mcr-1* among hospitalised patients decreased from 14.3% in 4498 patients in 2016 to 6.3% in 5657 patients in 2019 [26]. Our results are in line with other European studies. In the Netherlands, the prevalence of the *mcr-1* gene was found to be 0.35% in 576 patients [17], and in Paris (France) it was 0.58% out of 1217 patients from six hospitals [16]. The *mcr-1* gene was not detected in 330 hospitalised patients from Slovenia, 258 healthcare workers from Spain, 653 patients from a university hospital in Western France or in 1144 healthy individuals and inpatients in Switzerland [13,14,15,27].

However, the above-mentioned studies did use different screening methodologies. Whilst some of the studies tested the samples using direct PCR detection [17] or enriched broths followed by selective culture of the PCR-positive samples [9,26], most of the European studies used a selective culture followed by a PCR or WGS detection of the *mcr*-genes in colistin-resistant isolates [13,14,15,16,27]; and none of the studies tested for the presence of the *mcr-6* to *-8* genes.

It is important to note that cultures on selective media supplemented with colistin (3.5 mg/L) resulted in the detection of 294 isolates; however, only 131 were confirmed to be colistin-resistant using the broth microdilution method (MIC >2 mg/L). In addition, the growth of colistin-susceptible isolates on selective media supplemented with colistin has been described in other studies [16,27]. This is probably caused by the poor diffusion of colistin through the agar, and it underlines the need to confirm colistin resistance in an isolate cultured on this type of media by broth microdilution.

In our study, only four of the 131 colistin resistant isolates carried the *mcr-1* gene; the underlying mechanism of colistin resistance was not investigated in any of the remaining isolates. However, a higher MIC value was found in a majority of these isolates and is a characteristic of the resistance caused by chromosomal mutations affecting the synthesis of lipopolysaccharide (LPS), e.g., mutations in the two-component systems, PmrAB and PhoPQ, or in the regulatory protein MgrB that regulates modification of LPS [1].

In our study, exposure to low concentrations of colistin (0.25 mg/L and higher) led to a reactivation of the *mcr-1* gene after the excision of the IS2 element from its promoter region in *mcr-1* positive/colistin- susceptible *E. coli*. As we did not find the target site duplications after the IS2 excision, we think the precise excision of IS2, which was essential for the restoration of MCR-1 production, happens via a non-replicative transposition. Similar to our study, the precise excision of IS203v (which belongs to the same IS3 family as IS2 in this study) was described in *E. coli* O157:H7 where the Shiga toxin 2 was reversibly inactivated by IS203v [28]. The changes in MICs after culture with low concentrations of colistin have already been described in several isolates, including *E. coli* carrying the *mcr-9* gene [29]; *Shigella sonnei* that was susceptible due to the inactivation of the *mcr-1* gene through a 22 bp duplication within the gene [30]; and *E. coli* carrying an inactivated *mcr-1* gene by the insertion of an IS*1294b* element [31]. A further two studies also reported an inactivated *mcr-1* gene in *E. coli*; however, the inducibility of colistin resistance was not tested [32] or found to be unlikely due to the character of the genetic change [17].

Before our study, the detection of the *mcr-1* and *mcr-4* genes was reported in Czech clinical human isolates. From the 610 isolates collected between 2008 and 2018, eight (1.3%) *mcr-1* and two (0.3%) *mcr-4* positive isolates were found [33]. Unfortunately, the WGS data on isolates from this study are not available.

Another two human *mcr-1 E. coli* isolates were detected through an investigation of 177 stool samples of Czech travellers or expatriates residing temporarily in the Czech Republic. The isolates had different STs to the isolates detected in our study [34].

More importantly, the high prevalence of the *mcr-1* gene (70.6 % and 21%) was found recently in two studies investigating imported and domestic raw, mainly poultry, meat products retailed in the Czech Republic [20,25]. It is interesting that the same *E. coli* sequence types, 744 and 69, which were identified in turkey meat imported to the Czech Republic from Poland, Brazil, Germany, and in turkey meat that originated in the Czech Republic [25], were also identified in our study in hospitalised patients. Furthermore, all the ST744 and ST69 isolates from the above-mentioned study carried the *mcr-1* on the same IncX4 plasmid type.

## 4. Materials and Methods

### 4.1. Samples

Between June 2018 and September 2019, rectal swabs or faecal samples from patients (both adult and paediatric) hospitalised at the Motol University Hospital, Prague, were investigated. This hospital is a tertiary care hospital with 2200 beds and approximately 80,000 hospitalisations per year.

### 4.2. Screening for the mcr-Mediated Colistin Resistance

The rectal swabs or faecal samples were enriched in 5 mL Enterobacteriaceae enrichment broth (Mossel, Oxoid, UK) overnight at 37 °C. For direct detection of the *mcr* genes, DNA was extracted from 1 mL of the enriched cultures using a DNeasy^®^ Blood & Tissue Kit (Qiagen, Germany). The *mcr*-genes *1* to *8* were detected by two SYBR^®^ Green (Qiagen, Germany) based multiplex qPCR assays (CFX96 instrument, Bio-Rad Laboratories, Hercules, CA, USA), respectively. The primers and sequences for the synthetic DNA positive controls (GeneArt Strings, Thermo Fisher Scientific, Bremerhaven, Germany) were derived from previous studies reporting the *mcr-1* to *-8* genes [3,35,36,37,38,39,40,41] and are listed in Supplementary Table S3.

The enriched cultures were inoculated in parallel onto selective chromogenic agar Brilliance UTI Clarity agar (Oxoid, United Kingdom) supplemented with colistin (3.5 mg/L). Concentration of colistin was chosen on the basis of selective media developed by Nordmann et al. [21]. Suspected Enterobacteriaceae were identified using MALDI-TOF/MS Biotyper v 3.1 (Bruker Daltonics, Belgium). The isolates of intrinsically colistin-resistant Enterobacteriaceae (e.g., *Proteus*, *Morganella*, *Providentia*, etc.) were excluded from further analyses. Resistance to colistin was confirmed using the broth microdilution (BMD) method (Erba Mannheim, Germany), and a breakpoint of >2.0 mg/L was applied as recommended [42]. The susceptible *E. coli* ATCC 25922 and the colistin-resistant *mcr-1* positive *E. coli* NCTC 13846 strains were used as a quality controls.

The colistin-resistant isolates of Enterobacteriaceae were tested for the presence of the *mcr*-genes as described above, and the *mcr*-positive isolates were characterised by whole-genome sequencing (WGS).

In the case of the *mcr-1* positive enrichment by PCR, but negative culture on chromogenic selective media supplemented with colistin, the enrichment was inoculated on chromogenic media without colistin, and the grown colonies of Enterobacteriaceae were tested for the presence of the *mcr*-genes. The *mcr*-positive isolates were characterised as described above.

### 4.3. Whole-Genome Sequence Data Analysis of mcr-Positive Isolates

Short read sequencing was performed on a MiSeq sequencer (Illumina) after DNA sequencing library preparation using a Nextera XT DNA Library Preparation Kit (Illumina). For long read sequencing, a MinION NanoPore DNA technology (Oxford Nanopore Technologies (ONT) was applied. MinION libraries were prepared with a Ligation Sequencing Kit, #SQK-LSK109 (Oxford Nanopore Technologies (ONT)) and sequenced in a #FLO-MIN106 flow cell. Fast5 read files were base-called and converted to fastq format using Guppy v3.0.3+7e7b7d0 (ONT). The hybrid assembly of long and short reads was done using Unicycler v0.4.7 [23].

Bioinformatic analyses were performed using software tools available from the Centre for Genomic Epidemiology websites (https://cge.cbs.dtu.dk, accessed on 10 November 2020). MLST (Achtman and Pasteur) and core genome (cg) MLST were determined by MLST 2.0 and cgMLSTFinder 1.1 [43]. Serotype was defined by SerotypeFinder 2.0 [44]. Acquired antimicrobial resistance genes and mutations associated with antimicrobial resistance were identified using ResFinder 3.2 [45]. Plasmid classification was performed using PlasmidFinder 2.0 [46]. The phylogroup was determined using ClermonTyper [47], and IS elements were identified by ISfinder and ISsaga [48]. The sequence similarity between plasmid sequences was assessed by MUSCLE alignment using Geneious software v10.2.6.

### 4.4. Colistin Resistance Induction Assay in a mcr-Positive Colistin-Susceptible Isolate

The inducibility of colistin resistance was tested in the colistin-susceptible *mcr-1* positive *E. coli* isolate. Briefly, the *mcr-1* positive/colistin-susceptible *E. coli* isolate was cultured overnight on non-selective medium (Columbia blood agar). A bacterial suspension in saline (100 µL) adjusted to turbidity of 0.5 McFarland was inoculated, in triplicate, into 5 mL of LB broth (Luria Bertani, Oxoid, UK) containing colistin at concentrations of 0, 0.064, 0.125, 0.25, 0.5, and 1 mg/L and grown at 37 °C with an agitation of 200 rpm overnight (18 h). The culture grown in the LB broth was centrifuged using 4000 rpm for 10 min, and the minimum inhibitory concentration (MIC) to colistin was determined directly from the bacterial pellet according to the manufacturer’s instructions (Erba Mannheim, Germany).

### 4.5. Confirmation of MCR-1 Expression

To confirm the production of MCR-1 phosphoethanolamine transferase in the *mcr-1* gene positive isolates, the NG-Test MCR-1 lateral flow immunoassay (Abbott, USA) was used according to the manufacturer’s instructions.

### 4.6. Antimicrobial Resistance Profile Determination

In addition, for the *mcr*-positive isolates, the MICs for 24 commonly used antimicrobials in Enterobacteriaceae infections were determined by the microdilution method (Erba Mannheim, Germany) using EUCAST breakpoints [42]. The Clinical laboratory standards institute (CLSI) breakpoints were used for tetracycline, cefoperazone and netilmicin, as the EUCAST does not define the values [49].

### 4.7. Comparative Analysis of Czech mcr-1 E. coli Isolates from Humans and Food of Animal Origin

To investigate the genetic relatedness between Czech *mcr-1* positive isolates from humans (this study) and food of animal origin [25], the sequences of isolates revealing the same sequence type, and which carried the *mcr-1* gene in the same plasmid type, were compared using core genome MLST (cgMLST) analysis comprising 2513 loci for *E. coli* in Ridom Seqsphere+ v7.2.0 (Ridom GmbH, Münster, Germany). Subsequently, a minimum spanning tree (MST), ignoring missing values, was constructed. The threshold cluster identification was 10 alleles according to the Ridom SeqSphere+ software.

In order to find genomes closely related to the *mcr*-positive isolates in this study, the BacWGSTdb 2.0 was used to perform the genomic epidemiological analysis [50].

## 5. Conclusions

To the best of our knowledge, this is the first study on the intestinal carriage of the *mcr-1* to *-8* genes in hospitalised patients. A low prevalence of the *mcr-1* gene was identified, and the other *mcr*-genes were not detected. The *mcr-1* positive *E. coli* isolates were genetically distinct; the IncX4 plasmid type was the most frequent *mcr-1* gene vector. Our results indicate that, currently, *mcr*-mediated colistin resistance is of little threat to patients in Czech hospitals. The genetic relatedness of human and raw turkey meat *mcr-1 E. coli* isolates suggests that the dissemination of the *mcr-1* gene has a possible link to the domestic production of retailed meat, but as to whether the source is of human or animal origin, or through meat contamination, needs to be investigated further. In terms of clinical practice, it should be highlighted that colistin resistance can be inducible in clinical isolates that carry the *mcr-1* gene disrupted by IS element insertions.

## Figures and Tables

**Table 1:** Colistin minimal inhibitory concentration distribution in Enterobacteriaceae isolates grown on selective agar.

Species	Resistant > 2 mg/L (EUCAST)				
	≤2 mg/L ^E^	4 mg/L	8 mg/L	≥16 mg/L	Total Resistant
*Escherichia* spp. (*n* = 84) ^A^	66	4	4	10	18
*Klebsiella* spp. (*n* = 132) ^B^	53	7	11	61	79
*Enterobacter* spp. (*n* = 47) ^C^	17	−	1	29	30
*Citrobacter* spp. (*n* = 22) ^D^	20	−	2	−	2
*Salmonella* spp. (*n* = 9)	7	−	2	−	2
Total (*n* = 294)	163	11	20	100	131

^A^ Including *E. coli* (*n* = 83) and one isolate of *E. hermannii*. ^B^ Including *K. pneumoniae* (*n* = 112); *K. variicola* (*n* = 15); *K. oxytoca* (*n* = 3); *K. aerogenes* (formerly *Enterobacter aerogenes*) (*n* = 2). ^C^ Including *E. cloacae* (*n* = 26); *E. kobei* (*n* = 16); *E. asburiae* (*n* = 4); *E. ludwigii* (*n* = 1). ^D^ Including *C. freundii* (*n* = 13); *C. braakii* (*n* = 4); *C. koseri* (*n* = 2); and one isolate each for *C. amalonaticus*, *C. farmeri*, *C. murliniae*. ^E^ The probable causes of the growth of colistin-susceptible bacteria on selective agar are i) non-homogenous distribution in agar; ii) binding of the drug to the plastic of the Petri dish; and iii) a high amount of susceptible bacteria in the inoculum [21].

**Table 2:** The minimal inhibitory concentration (mg/L) of colistin and other antimicrobials with a detected resistance in the *mcr-1* positive *E. coli* isolates.

Isolate No.	COL (≥4)	AMP (≥16)	AMS (≥16/4)	CFZ (≥8)	CXM (≥16)	CPZ (≥64)	PIP (≥32)	TET (≥16)	TGC (≥1)	T/S (≥8/152)	CIP (≥1)	TOB (≥4)	CMP (≥16)
P642A	4	>128	16/8	16	16	8	>128	>32	1	>4/76	0,5	1	8
P732A	8	>128	128/64	16	8	>64	>128	>32	0.25	>4/76	>8	4	4
P1301A	0.25	2	2/1	2	4	<0.5	<1	>32	0.25	0.25/4.75	8	1	>32
P1519A	8	>128	64/32	8	4	2	>128	>32	0.5	>4/76	>8	1	8

MIC was determined by broth microdilution (BMD) for ampicillin (AMP); ampicillin/sulbactam (AMS); cefazolin (CFZ); cefuroxime (CXM); aztreonam (AZT); gentamicin (GEN); amikacin (AMK); colistin (COL); trimethoprim/sulfamethoxazole (T/S); ciprofloxacin (CIP); chloramphenicol (CMP); tetracycline (TET); piperacillin (PIP); piperacillin/tazobactam (PIT); cefotaxime (CTX); ceftazidime (CAZ); cefoperazone (CPZ); cefoperazone/sulbactam (CPS); cefepime (CEP); meropenem (MER); ertapenem (ERT); tigecycline (TGC); netilmicin (NET); tobramycin (TOB). The breakpoints for resistance in mg/L are according to EUCAST (Clinical breakpoints v.10) and are in brackets. For tetracycline, netilmicin and cefoperazone, CLSI break-points were used since EUCAST does not define these values. All isolates were susceptible to amikacin, aztreonam, piperacillin/tazobactam, cefotaxime, ceftazidime, cefoperazone/sulbactam, cefepime, gentamicin, meropenem, ertapenem and netilmicin.

**Table 3:** The epidemiological characteristics of *mcr-1* positive samples/isolates.

Isolate/Sample	Isolation (mm/yyyy)	Dept.	ST Achtman/Pasteur	cgMLST	Serotype	Phylogroup (Clermont Typing)
P224 *	8/2018	Children neurology	−	−	−	−
P642A	10/2018	Pneumology	8778/76	81202	O9:H10	A
P732A	11/2018	Anesthesiology and ICM	69/3	71872	O unknown: H18	D
P1301A	5/2019	Children cardiology	744/2	58727	O unknown: H9	A
P1519A	7/2019	Children surgery	1193/53	31972	O18a:H5	B2

* In sample P224, the *mcr-1* gene was detected by PCR and confirmed by Sanger sequencing; it was not possible to culture the isolate carrying the *mcr-1* gene.

**Table 4:** Localisation of antimicrobial resistance (AMR) genes and mutations and virulence genes in genomes and plasmids of *mcr-1* positive *E. coli* isolates.

Isolate	Contig ^A^	Size (bp)	Plasmids (PlasmidFinder 2.0)	AMR Genes (ResFinder 3.2)	AMR Mutations (ResFinder 3.2)	Virulence Genes (VirulenceFinder 2.0)
P642A	1	4,726,579	none (chromosome)	*mdf*(A)	none	*gad; ireA; iss*
	2	129,386	IncFIB(AP001918); IncFII; IncQ1	*aadA1*; *aph*(3″)-Ib; *aph*(6)-Id; *bla*_TEM-1B_; *sul1*; *sul2*; *tet*(A); *dfrA1*	none	*iroN; iss*
	3	47,696	IncX1	*bla*_TEM-1B_; *qnrS1*	none	none
	4	33,308	**IncX4**	*mcr-1.1*	none	none
P732A	1	5,262,918	none (chromosome)	*mdf*(A)	*parE* p.S458A (FQ); *parC* p.S80I (FQ); *gyrA* p.S83L (FQ); *gyrA* p.D87N (FQ)	*air; eilA; gad; iss; lpfA*
	2	93,793	not found	none	none	none
	3	85,283	IncFIA; IncFIB(AP001918); IncQ1	*aac*(3)-IId; *aph*(3″)-Ib; *aph*(6)-Id; *catA1*; *sul2*; *tet*(B)	none	none
	4	34,080	**IncX4**	*mcr-1.1*	none	none
	5	14,745	not found	*aadA5*; *bla*_TEM-1B_; *mph*(A); *sul1*; *dfrA17*	none	none
	6	8431	not found	*aph*(3′)-Ia	none	none
P1301A	1	4,778,337	IncQ1 ^B^ (chromosome)	*aph*(3″)-Ib; *aph*(3′)-Ia; *aph*(6)-Id; *mdf*(A); *catA1*; *sul2*; *tet*(B)	*parC* p.A56T; *parC* p.S80I; *gyrA* p.S83L; *gyrA* p.D87N	*gad*
	2	89,356	IncFIA;IncFIB(AP001918); IncFII(pCoo)	none	none	none
	3	73,158	IncFII(pCoo)	*tet*(B)	none	none
	4	34,639	**IncX4**	*mcr-1.1*	none	none
P1519A	1	5,082,995	none (chromosome)	*mdf*(A)	*gyrA* p.S83L; *gyrA* p.D87N; *parC* p.S80I; *parE* p.L416F	*gad; iha; sat; vat*
	2	250,486	**IncHI2; IncHI2A**	*mcr-1.1*; *aadA1*; *aadA2b*; *aph*(3′)-Ia; *cmlA1*; *sul3*; *tet*(A)	none	none
	3	110,729	Col156; IncFIA; IncFIB(AP001918); IncQ1	*aph*(3″)-Ib; *aph*(6)-Id; *bla*_TEM-1B_; *mph*(A); *sul2*; *tet*(B); *dfrA17*	none	*senB*
	5	2113	Col(BS512)	none	none	none

^A^ The hybrid assembly of long and short reads was done using Unicycler v0.4.7. [22]. ^B^ The detection of IncQ1 presence in contig no. 1 corresponding to the genome of *E. coli* indicates plasmid integration into the chromosome of the host in isolate P1301A. The plasmids carrying *mcr-1.1* gene are in bold.

## Data Availability

Part of the data were previously reported at the 30th European Congress of Clinical Microbiology and Infectious Diseases, 18–21 April 2020, Paris, France (Abstract no. 4183). The datasets used and/or analysed during the current study are available from the corresponding author on reasonable request.

## Supplementary Materials

The following are available online at https://www.mdpi.com/2079-6382/10/3/258/s1, Table S1: Detailed annotation of plasmid IncX4-P1301A, Table S2: Genomic epidemiological analysis of relationship of *mcr*-positive isolates to available published genomes (based on cgMLST strategy), Table S3: The primers used to detect the *mcr-1* to *mcr-8* genes.
